# A novel *Salmonella* strain inactivated by a regulated autolysis system and expressing the B subunit of Shiga toxin 2e efficiently elicits immune responses and confers protection against virulent Stx2e-producing *Escherichia coli*

**DOI:** 10.1186/s12917-017-0962-2

**Published:** 2017-02-01

**Authors:** Gayeon Won, Tae Hoon Kim, John Hwa Lee

**Affiliations:** College of Veterinary Medicine, Chonbuk National University, Iksan campus, Gobong-ro 79, Iksan, 54596 Republic of Korea

**Keywords:** Shiga toxin, Edema disease, Bacterial ghost, *Salmonella* typhimurium

## Abstract

**Background:**

*Salmonella* Typhimurium (*S.* Typhimurium) inactivated by a regulated autolysis system was genetically engineered to express the homo-pentameric B subunit of Shiga toxin 2e (Stx2eB) on its surface. To prepare a strain able to yield autolyzed *Salmonella* bearing Stx2eB, the plasmid pJHL184 harboring *stx*_*2eB*_ gene was transformed into the attenuated *S.* Typhimurium strain, JOL1454. Stx2eB subcloned into the antigen delivery cassette of the plasmid was expressed as fusion protein with the outer membrane protein

**Results:**

The expression of Stx2eB fused to the signal peptide in JOL1454 was validated by immunoblot analysis. To determine the immunogenicity of JOL1454, female BALB/c mice were intramuscularly injected with 1 × 10^8^ CFU of the inactivated cells at weeks 0 and 2. Significantly elevated levels of IgG and IgA specific to Stx2eB was observed at weeks 4 and 6 post-immunization (PI) (*P* <0.05). Proportion of CD3^+^CD4^+^ T lymphocyte subpopulation was also significantly augmented in in vivo stimulated splenocytes relative to that in the control group. The increased titers of IgG1 and IgG2a, and of immunomodulatory cytokines indicated that the immunization elicited Th1 and Th2 immune responses. Further, immunomodulatory cytokine genes (IL-6, IL-17A, IL21 and JOL1454) efficiently upregulated in naïve porcine peripheral blood mononuclear cells (PBMCs) pulsed with JOL1454. At week 6 PI, following the challenge with a virulent Stx2e-producing *Escherichia coli* in the mice, all immunized mice survived whereas approximately 30% of the mice in the control group died.

**Conclusions:**

JOL1454 provided superior immunogenicity and effective protection against challenge with a sublethal dose, which demonstrates its potential as a candidate vaccine against edema disease.

## Background

Among Shiga toxin-producing *Escherichia coli* (STEC) strains, Stx2e is the second most common subtype of *stx*_*2*_ found in isolates from environmental sources [1]. Although STEC harboring the *stx2e* gene has only rarely been detected in human feces, causing mild diarrhea [2], *stx2e* is the most frequently encountered variant of *stx* gene in STEC isolated from porcine feces [3], and STEC harboring the *stx2e* gene has been reported to contribute to the virulence of edema disease (ED) in weaned piglets [4]. Shiga toxin 2e, encoded by the *stx2e* gene, inhibits protein biosynthesis by ribosome inactivation, which is chiefly responsible for the clinical signs and lesions of ED, including subcutaneous and submucosal edema. In addition, brain vascular injury caused by endothelial cell edema can elicit fatal neurological disorders and sudden deaths [4]. Binding of the non-toxic pentamer B subunits of Stx2e (Stx2eB) to the cell surface globotetraosylceramide (Gb4Cer) receptor allows the toxic A subunit of Stx2e (Stx2eA) to enter the cytoplasm, where cytotoxic effects occur [5]. Prevention of Stx2eB binding to the relevant receptors located on the intestines and cerebral endothelial cells thus represents a possible mechanism to prevent the transmission of this pathogen. Hence, Stx2eB may represent a practical target for the generation of neutralizing antibodies that could contribute to impairing the interaction between Stx2eB and cell surface receptors on intestines, thereby inhibiting the subsequent cytotoxic effects on epithelial cells that are mediated by Shiga toxin [5].

ED causes significant economic losses due to sudden deaths of infected pigs. The factors affecting the prevalence of ED are not clearly understood, despite mortality rates due to ED as high as 50 to 90% [6], with substantial variance among countries and farming units, and depending on the health status of the infected pigs. Elaborate efforts have been made in an attempt to reduce disease burden and economic loss in the swine industry. In particular, the need for optimal vaccination strategies against ED has increased, as frequently reported incidences of antimicrobial-resistant STEC in swine farms worldwide become progressively more burdensome to public health [7]. In addition, the administration of antibiotics appears to come too late to treat diseased pigs, since even when antibiotics are administered at the onset of visible clinical signs, severe neurological symptoms subsequently develop. Accordingly, several vaccine strategies based on targeting Shiga toxin have arisen. Active and passive immunization of piglets with an Stx2e toxoid has been reported to provide protection against challenge with Stx2e toxin [6]. However, those results did not imply that the toxoid would protect piglets against virulent STEC infection. Live attenuated STEC carrying genetically modified Stx2e has been constructed that confers somewhat effective protection against a challenge with a lethal dose [6], although a high degree of reactogenicity remained. Thus, despite the continuous effort to improve vaccines against ED, no commercial vaccine is currently available.

A virulent strain of *Salmonella* Typhimurium has been successfully prepared for use in expressing a broad range of homologous antigens, to induce enhanced immune responses against them [8]. To minimize the risk of live attenuated *Salmonella* reverting to a virulent strain, autolyzed ghost strains derived from *Salmonella enterica*, including *S*. Enteritis [9], *S*. Gallinarum [10], and *S*. Typhi [11] have been genetically engineered for use as candidate vaccines or vaccine delivery carriers. Inactivated bacterial ghosts retaining the entire surface antigenic features of the original bacteria can efficiently target antigen-presenting cells and induce strong immunological responses [12].

A particular form of autolyzed bacteria generated via PhiX174 *E* gene-mediated lysis, so-called “bacterial ghosts” (BGs), are non-living gram-negative bacterial cell envelopes that lack cytoplasmic contents, yet conserve all the surface components of their parental bacteria [12, 13]. BGs have induced strong immunological immune responses against retained surface antigenic determinants, such as lipopolysaccharide and peptidoglycan [14]. The capacity of BGs as a presentation system for heterologous antigens has been evaluated in previous studies [15, 16]. Foreign target proteins presented by BGs have been successfully expressed as outer membrane proteins via fusion with signal sequences, or been translocated into the periplasmic space [16]. *Salmonella* ghost strains have been widely used as vehicles for antigen delivery due to their ability to induce adjuvant effects by invading host immune systems [15]. In the present study, an attenuated *S*. Typhimurium strain harboring a recombinant plasmid carrying a lysis gene permitting induction of bacterial autolysis, as well as the *stx*_*2eB*_ gene, was constructed. Lysis of *S*. Typhimurium was mediated by the PhiX174 *E* gene under the control of the face-to-face promoter system to generate *S*. Typhimurium ghost cells [17]. During lysis, Stx2eB protein fused with the outer membrane protein A signal peptide (*ompA* ss) is expressed, which enables Stx2eB to be exported across membranes of autolyzed cells. The immunogenicity of *S.* Typhimurium ghosts expressing Stx2eB was evaluated in a mouse model, and protective efficacy was also examined by challenging immunized mice with virulent STEC.

## Methods

### Bacterial strains and culture conditions

All bacteria strains and plasmids used in this study are described in Table 1. The *S.* Typhimurium mutant strains, Δ*asd* Δ*lon* Δ*cpxR* JOL912 and Δ*asd Escherichia coli* χ6212, were grown in either Luria-Bertani (LB) broth or LB agar at 37 °C with 50 μg/ml of diaminopimelic acid (DAP) (Sigma-Aldrich, St. Louis, MO). The bacterial strains harboring the ghost plasmid were grown at 28 °C in nutrient broth (NB) containing 0.2% L-arabinose. All bacterial strains were stored at −80 °C in growth medium containing 20% glycerol.

**Table 1:** Bacterial strains and plasmids used in this study

Strain/plasmid	Description	Reference/source
Bacterial strains		
*E. coli*		
BL21(DE3)pLysS	F^−^ *ompT hsdSB* (rB^−^ mB^−^) *dcm galλ*(DE3) pLysS Cmr	Promega
JOL232	F^−^ λ^−^ ϕ80 Δ(*lacZYA-argF*) *endA1 recA1 hadR17 deoR thi-1 glnV44 gyrA96 relA1 ΔasdA4*	Lab stock
JOL606	Wild-type LT^+^, K99^+^, F6^+^,F18^+^, *stx*_*2*_^+^, *stx*_*2e*_^+^ STEC isolate from pig	Lab stock
JOL654	Wild-type LT^+^,F18^+^,STa^+^, *stx*_*2*_^+^, *stx*_*2e*_^+^ STEC isolate from pig	Lab stock
*S*. Typhimurium		
JOL912	*Δlon ΔcpxR Δasd*, a derivative of S. Typhimurium	[20]
JOL 1400	JOL912 harboring pJHL184	This study
JOL 1454	JOL912 harboring pJHL184-*stx*_*2eB*_	This study
Plasmids		
pET28a	IPTG-inducible expression vector; Km^r^	Novagen
pET28a-*stx*_*2eB*_	pET28a derivative containing *stx*_*2eB*_	This study
pJHL184	*asd* ^+^ vector, pBR ori plasmid carrying ss *ompA*/His_6_, multiple cloning site, cI857/λPR promoter, araC P_araBAD_, *phi*X174 lysis gene *E*	[47]
pJHL184-*stx*_*2eB*_	pJHL184 harboring *stx*_*2eB*_ gene	This study

### Construction of *Salmonella* ghosts bearing Stx2eB

The *S.* Typhimurium mutant strain Δ*asd* Δ*lon* Δ*cpxR*, JOL912, was prepared by allelic exchange methods as previously described [18]. The ghost plasmid, pJHL184, carries a pBR origin, a multiple cloning site (MCS), a C-terminal His-tag, and a ghost cassette [19]. The *stx*_*2eB*_ gene was amplified by polymerase chain reaction (PCR) from wild-type STEC JOL606 isolated from pig diarrhea, using the primer pair, S1: 5’-ccgccaattcaagaagatgtttatggcg-3’ and S2: 5’-ccgcaagcttgtcattattaaactgcac-3’. The thermal cycle parameters of the PCR reaction consisted of an initial denaturation at 94 °C for 5 mins, followed by 30 cycles of 95 °C for 15 s, 54 °C for 15 s, and 72 °C for 30 s, with a final extension step of 7 mins at 72 °C. The gene fragment was designed to produce an EcoRI restriction endonuclease site at the 5’ end and a HindIII site at the 3’ end. The resultant PCR products were subcloned into the overexpression plasmid pET28a, thus generating pET28a-*stx*_*2eB*_. The *E. coli* BL21 (DE3) pLysS strain was transformed with pET28a-*stx*_*2eB*_. Stx2eB protein was purified as described previously [19]. The *stx*_*2eB*_ gene-containing DNA fragments derived from pET28a-*stx*_*2eB*_ were subcloned into EcoRI/HindIII-digested pJHL184 to generate the recombinant plasmid pJHL184-*stx*_*2eB*_ [17]. Stx2eB secretion into the periplasmic space is achieved by fusion with the signal sequence of *E. coli* outer membrane protein OmpA, which was subcloned into the MCS of pJHL184. pJHL184-*stx*_*2eB*_ was initially transformed into *E. coli* χ6212 (JOL232) to maintain the stability of the plasmid in the absence of antibiotics, and the plasmid subsequently was introduced into JOL912 by electroporation. The resultant strain was designated as JOL1454. The Δ*asd* Δ*lon* Δ*cpxR S.* Typhimurium strain JOL1400, carrying pJHL184, was used as a vector control.

### Production of *Salmonella* ghosts bearing Stx2eB

A single colony of JOL1454 was inoculated into nutrient broth containing 0.2% L-arabinose, and the inoculum was incubated at 28 °C with agitation at 120 rpm until mid-logarithmic growth phase to achieve mass production of the strain. The cells were collected and washed twice with nutrient broth (NB) to remove L-arabinose. The cells were resuspended in 100 ml NB and incubated at 42 °C in a shaking incubator at 200 rpm to induce *E* gene-mediated lysis over the course of 48 h. After lysis, the ghost cells were harvested via centrifugation at 13,000 rpm for 15 min, washed twice with sterile phosphate-buffered saline (PBS) (pH 7.4), and stored at −70 °C.

### Stx2eB expression in JOL1454

Western blot analysis was performed to verify the expression of Stx2eB antigen from JOL1454, as previously described [19]. Bacterial ghosts expressing the target antigen, and hyperimmune rabbit serum raised against Stx2eB, were prepared as previously described [19]. Protein lysates of the prepared ghost cells (5 μl) were subjected to sodium dodecyl sulfate-polyacrylamide gel electrophoresis (SDS-PAGE) on 15% gels. Resolved proteins were transferred onto polyvinylidene fluoride membranes (Millipore, Billerica, MA, USA), immunoblot analysis was performed, and immunoreactive bands were detected as previously described using hyperimmune rabbit serum raised against Stx2eB (1:5,000) and a HRP-labeled goat anti-rabbit IgG (1:8,000) [19]. JOL1400, JOL912 carrying pJHL184 and the purified Stx2eB protein were utilized as negative and positive controls, respectively. Subsequently, the amount of Stx2eB expressed in JOL1454 was relatively quantified in a calibration standard using indirect enzyme-linked immunosorbent assays (ELISA) [21]. Stx2eB containing 6 × His-tag at the C-terminal end in JOL1454 were purified by Ni–NTA spin columns (Ni–NTA Spin Kit, Qiagen) according to the manufacturer’s instruction. The His-tagged Stx2eB protein in a volume of 5 ml of the prepared ghost cell (1 × 10^8^ cfu per ml) were eluted from the column with 2 ml of elution buffer (7 M urea: 7 M urea; 0.1 M sodium dihydrogen phosphate; 0.01 M Tris · Cl; pH 8.0). A calibration standard was generated by using twofold serial dilutions of Stx2eB protein extracted from BL21 harboring pET28a-*stx*_*2eB*_. The diluted protein samples ranging from 1 μg to 31.25 ng, and the purified His-tagged protein as antigen were coated in the ELISA plate (Greiner) and incubated at 4 °C overnight. After the incubation, the hyperimmune rabbit serum raised against Stx2eB (1:300) and a HRP-labeled goat anti-rabbit IgG (1:5,000) were used as primary and secondary antibodies, respectively as previously described [19]. The concentration of the His-tagged Stx2eB protein expressed in JOL1454 were calculated by the known concentration of the purified Stx2eB corresponding to values of optical density at 490 nm in the calibration standard. The final concentration of the protein presented was based on the number of cells in initial bacterial culture.

### Animal experiments

Eighteen female BALB/c mice were randomly assigned to two groups at five weeks of age. The mice in group A were intramuscularly immunized with 1 × 10^8^ JOL1454 ghost cells at weeks zero and two. PBS was injected into the mice in group B, which served as a non-immunized control group. For measurement of total serum immunoglobulin (Ig) G, as well as of IgG1 and IgG2a, serum samples were collected, and for measurement of secretory IgA (sIgA), vaginal wash samples were collected, as previously described [20], at weeks 0, 2, 4 and 6. All samples were stored at −70 °C until used. For immunological assays using the primed splenocytes, additional 10 mice (5 mice per group) were inoculated at week 0 using the same protocol described above. Further, although ED results from oral transmission of the pathogen in natural infections, we found that ED did not occur in BALB/C model mice orally challenged with JOL654. However, intraperitoneal injection with the challenge strain efficiently induced symptoms in mice similar to those of ED such as hemorrhage in small intestine and bloody diarrhea. At week 7, non-immunized and immunized mice were intraperitoneally challenged with wild type STEC JOL654 isolated from porcine diarrhea (Table 1). The murine sublethal dose (2 × 10^7^ CFU) was determined by the Reed-Muench method [22] following a protocol used in a previous study [10]. Mice were monitored daily for mortality, clinical signs, and body weight for one week after inoculation.

### Humoral and cellular immunological response

Stx2eB-specific titers of total IgG, the IgG1 and IgG2a isotypes, and secretory IgA (IgA) in sera or vaginal washes, as appropriate, were measured by ELISA [19] on 96-well plates with 500 ng of purified Stx2eB protein coating each well. Fluorescence-activated cell sorting (FACS) was used to assay changes in T cell subpopulations induced by immunization. Splenocytes were aseptically isolated from mice at day seven post-immunization, and single-cell suspensions were prepared. One million cells were stained by combining them with anti-mouse CD3a-PE and anti-mouse CD4-perCP-vio700 antibodies (Miltenyi Biotec, Bergisch Gladbach, Germany) and incubating for 20 min. After washing twice with FACS buffer (Miltenyi Biotec, Bergisch Gladbach, Germany), changes in CD3^+^ and CD4^+^ T cell subpopulations were determined using a MACSQuant® analyzer (Miltenyi Biotec, Bergisch Gladbach, Germany). Splenocyte proliferation following antigen stimulation in vitro was assessed by incorporation of MTT (3-(4,5-dimethylthiazol-2-yl)-2,5-diphenyltetrazolium bromide), which is only converted to blue formazan dye by actively proliferating cells [23]. Splenocytes were isolated from the immunized group at week two post-immunization (PI). Suspensions of 1 × 10^6^ single cells were cultured in triplicate with 500 ng/ml of stx2eB antigen in RPMI 1640 medium (GIBCO, cat. no.11875093) containing 5% FCS (GIBCO, cat. no. 10099141), at 37 °C in a 5% CO_2_ incubator for 72 h. Following stimulation, the cells were incubated with MTT (1 mg/ml) for another 4 h. The precipitated blue formazan in each well was solubilized in dimethyl sulfoxide, and colorimetric absorbance was measured as optical density (OD) at 570 nm with a spectrophotometer. The results are presented as a stimulation index, calculated as the mean OD value of the wells stimulated with antigen, divided by the mean OD value of the unstimulated wells.

### Cytokine measurement

Induction of expression at the mRNA level of the cytokines, interleukin-4 (IL-4) and interferon-γ (IFN-γ), induced by immunization of the mice was measured by reverse transcription real-time PCR. At week 2 PI, splenocytes were isolated from immunized and non-immunized mice. Viable cells (1 × 10^6^) stimulated with 500 ng/ml of Stx2eB protein were incubated in a 96-well cell culture plate with RPMI 1640 medium supplemented with 20% fetal bovine serum (FBS) in a humidified 5% CO_2_ atmosphere for 72 h. Total RNA from the cultured cell suspensions was isolated using a GeneAll® Hybrid-R^™^ kit (GeneAll Biotechnology, Seoul, Korea) and converted into cDNA with a ReverTra Ace® qPCR RT Kit (FSQ-101, TOYOBO, Japan). Levels of IL-4 and IFN-γ mRNA expression were quantified by real-time reverse transcription polymerase chain reaction (RT-PCR). The sequences of the primer pairs used to amplify IL-4, IFN-γ and β-actin (used as an internal standard) were those described by Lut et al. [24]. The mRNA expression levels of the cytokines were determined by the threshold method, using Cycle threshold (ΔC_T_) values calculated based on the internal standard. The fold change of the mRNA levels compared to those of the non-immunized group is expressed as 2^-(ΔΔCT)^ [25].

### Cytokine profiles assessed in porcine PBMC pulsed with JOL1454

To determine the extent of porcine lymphocyte activation following stimulation of the lysed *S*. Typhimurium expressing Stx2eB, immunomodulatory cytokines were measured in mRNA level in naïve porcine peripheral blood mononuclear cells (PBMC) pulsed in vitro with JOL1454. Naïve porcine PBMCs were obtained from 2 ml of blood samples of five non-vaccinated pigs (15-18 kg, 6 weeks of age) raised at an experimental animal farm of the college by density gradient centrifugation using Histopaque-1077® solution (Sigma, St. Louis, MO) following the manufacturer’s instruction. After the PBMC isolation, the cells were placed in RPMI 1640 medium supplemented with 1% fetal bovine serum and 1% glutamine/penicillin/streptomycin (Thermo Scientific), and then plated at a density of 5 × 10^6^ per well in 96 well cell culture plate. Subsequently, the resuspended cells were treated with the lysed JOL1454 with 10 multiplicity of an infection (MOI), and were incubated for 48 h at 37 °C in a humidified atmosphere of 5% CO_2_. Following the incubation, the pulsed cells were lysed directly in 500ul of Trizol reagent for RNA extraction. The preparation of RNA and cDNA were carried out as described above and 2 μl synthesized cDNA were added as qRT-PCR template. Cytokine (IL-6, IL-17A, IL-21, IFN-γ) mRNA expressions were determined by using the primer pairs [26–28] with SYBR® Green Realtime PCR Master Mix (QPK-201, TOYOBO, Japan) following the manufacturer’s instruction. Porcine glyceraldehyde-3-phosphate dehydrogenase (GADPH) and 60s ribosomal protein L19 (RPL-19) genes were used as reference genes for normalization [29]. ΔC_T_ values were standardized by the average Ct values of two internal controls and non-stimulated cells. Relative fold changes were determined by 2^-ΔΔCT^ method [25].

### Statistical analysis

Data are presented as mean ± standard deviation (s.d.). One-way ANOVA test was used to evaluate significant differences in immune responses between the immunized and non-immunized groups. Statistical differences were considered significant when *P* values were <0.05.

## Results

### Construction of *Salmonella* ghosts expressing Stx2eB

**Fig. 1:**
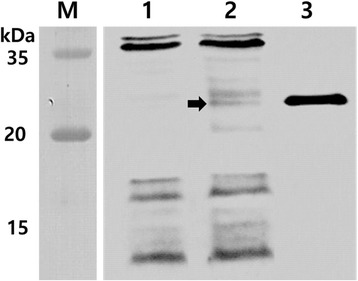
Western blot analysis of Stx2eB protein expressed in JOL1454 cells. The Stx2eB protein expressed and secreted by JOL1454 cells was detected by western blotting with rabbit anti-Stx2eB antibody. The arrow indicates the expected size of the fusion protein. JOL1400, consisting of JOL912 cells containing the empty vector pJHL184 and purified Stx2eB were used as a negative and positive control, respectively. Lane M, size marker; lane 1: vector control; lane 2: JOL1454; lane 3: purified Stx2eB

The *Stx2eB* gene was cloned under control of a constitutive promoter in the pJHL184 plasmid, which contains a convergent promoter system tightly regulating the induction of the *E* lysis gene and consequent lysis of JOL1454. To constitutively express *stx*_*2eB*_, JOL1454 cells were grown at 42 °C for 48 h in the absence of L-arabinose. Lysis of JOL1454 was confirmed by the inviability of cells plated from JOL1454 cultures grown at 42 °C. No viable cells were observed on LB plates after overnight incubation under conditions wherein the *E* gene was repressed (data not shown). The Stx2eB protein fused to ompA expressed in JOL1454 was verified by western blot analysis. The distinct immuno-reactive band of Stx2eB fused with OmpA was observed at ~24 kDa (Fig. 1; lane 1). Considering that the size of Stx2eB protein is ~13 kDa, we speculated that OmpA ss were properly fused with the target protein. In the lane 1 of Fig. 1 in which the lysed JOL1400 was loaded, the band corresponding to the size of Stx2eB was not shown. For the positive control, Stx2eB fused to 6xHis purified from pET28a-*stx2eB* in BL21 was loaded where immuno-reactive band was detected (Fig. 1; lane 3). The relative amount of Stx2eB protein expressed in JOL1454 was estimated by the linear regression standard curve showing a fit of *R* ^2^ = 0.971. Concentration of the His-tagged Stx2eB was approximately 909.18 ng per 10^8^ cfu of JOL1454.

### Humoral and mucosal immune responses raised by immunization

**Fig. 2:**
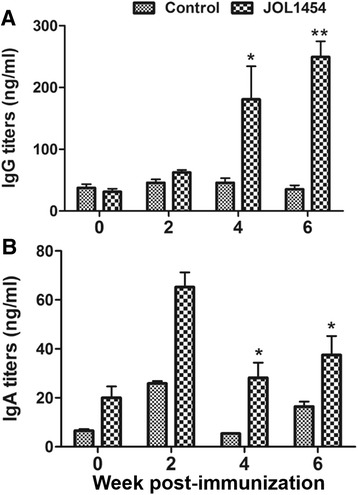
Stx2eB antigen-specific humoral immune responses in immunized and non-immunized mice. Titers of **a** serum IgG, and **b** secretory IgA in ng/ml. Data are presented as the means of all mice in each group (*n* = 9), and error bars indicate s.d. *, *P* < 0.05 and **, *P* < 0.001 vs. control group at each week post-immunization

**Fig. 3:**
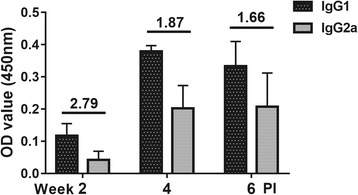
The levels of two IgG isotypes, IgG1 and IgG2a, elicited by JOL1454 immunization. The values indicate the means of optical density at 450 nm from sera collected from immunized mice (*n* = 9), and error bars indicate standard deviation (s.d). The numbers above the bars indicate the IgG1/IgG2a ratios. PI: post-immunization

The immunogenicity induced by JOL1454 immunization of the mice was assessed by measuring Stx2eB-specific serum IgG and sIgA titers. The sIgA and IgG titers specific to the Stx2eB antigen were significantly elevated (*P* <0.05) in the JOL1454 group compared to those in the control group (Fig. 2). The IgG titers significantly increased at week 4 (*P* <0.05, 2.59-fold) and week 6 post-immunization (PI) (*P* <0.001, 2.79-fold) compared to those measured in the negative control. The concentration of sIgA was moderately increased at week 2 PI and was significantly raised in comparison to those in the negative control at week 4 and 6 PI (*P* <0.05). Furthermore, the ratio of the IgG1 to IgG2a (IgG1/IgG2a) immunoglobulin isotypes, markers of T helper 2 (Th2) and T helper1 (Th1) lymphocytes, respectively, was calculated from the titers of the individual isotypes. JOL1454 immunization induced IgG1 to a greater degree than it did IgG2a (Fig. 3). The level of IgG1 was consistently higher than that of IgG2a throughout the entire observation period (Fig. 3).

### T lymphocyte proliferation induced by immunization

**Fig. 4:**
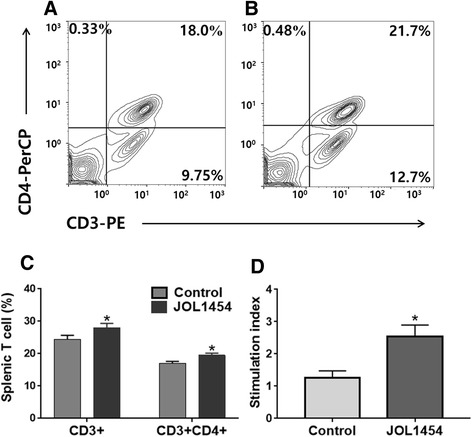
T cell-related immune responses elicited in JOL1454-immunized mice. Representative flow cytometry scatter dot plots for CD3+ and CD3 + CD4+ splenic T cell populations of non-immunized mice (**a**) and the immunized mice (**b**). The subpopulations are presented as a percentage of gated cells. CD3+ and CD3 + CD4+ T lymphocyte populations in immunized and non-immunized mice (**c**) and stimulation index (SI) values of splenic T cells of the purified Stx2eB protein-immunized group, determined by MTT assay (**d**). The values are expressed as the mean ± s.d. of five individual values. *, *P* < 0.05

Changes in splenic T cell fractions at week one post-immunization were assessed by measuring expression of the T cell surface markers CD3 and CD4 using flow cytometry. Both the CD3^+^ T cell population and CD3^+^CD4^+^ T cell subpopulations were significantly elevated (*P* <0.05, respectively) in JOL1454-immunized mice (Fig. 4a), leading to significant average increases of 3.52% and 2.6% in the CD3^+^ and CD3^+^CD4^+^ T cell subpopulations, respectively, compared to the non-immunized group. In the MTT cell viability assay, the JOL1454-immunized mice showed a significant increase (*P* <0.05) in stimulation index (SI) at week 2 PI, resulting in a significantly increased mean SI of 2.54 ± 0.49 in the immunized group, compared to 1.26 ± 0.29 in the PBS control group (Fig. 4b).

### Cytokine measurement

**Fig. 5:**
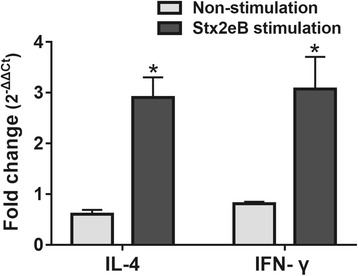
Cytokine mRNA transcript levels in stimulated and unstimulated splenic T cells isolated from JOL1454-immunized mice. The mRNA transcript levels of cytokines were evaluated by performing RT q-PCR with gene-specific primers. Each fold change value represents the mean ± standard error of the mean (SEM) of five individual values. *P < 0.05 when values were compared with the control group

To evaluate immunomodulatory cytokines secreted by the stimulated splenic lymphocytes, mRNA copy numbers of the IL-4 and IFN-γ cytokines were analyzed by qPCR. Fold changes were normalized against the murine β-actin gene. In vitro stimulation of splenic T cells with JOL1454 resulted in marked changes in relative fold values for the IFN-γ (*P* <0.05) and IL-4 (*P* = 0.01) cytokines in the immunized group compared to those in the control group (Fig. 5). These results indicated that the expression of IL-4 and IFN-γ in the immunized group were significantly upregulated compared to the control group, following stimulation of splenic T cells with Stx2eB.

### Immunomodulatory cytokines measured in the activated porcine PBMC

**Fig. 6:**
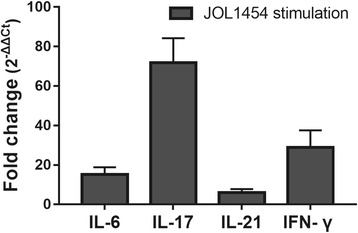
The mRNA expression of cytokines evaluated in in vitro stimulated porcine PBMC. The immunomodulatory cytokines were measured in mRNA level by using qRT PCR. Relative fold changes were calculated based on 2^-ΔΔCT^ method. Data are presented with the mean ± SEM (*n* = 5)

In vitro stimulation of porcine PBMCs with JOL1454 induced upregulation of immunomodulatory cytokine gene expressions (Fig. 6). The expression of IFN-γ, one of Th1-specific cytokine [29] and IL-6 promoting Th2 responses [30] increased up to 28.9 ± 8.5 and 15.13 ± 3.7 fold, respectively. Gene expression of IL-17A involving with differentiation of Th17 cells [31], were predominantly upregulated in the stimulated PBMC (71.88 ± 12.29-fold). Concomitantly, a moderate increase of expression of IL-21 produced by Th17 cells [32, 33] (6.07 ± 1.73-fold) were also observed in the pulsed PBMCs.

### Protective efficacy conferred by JOL1454

**Fig. 7:**
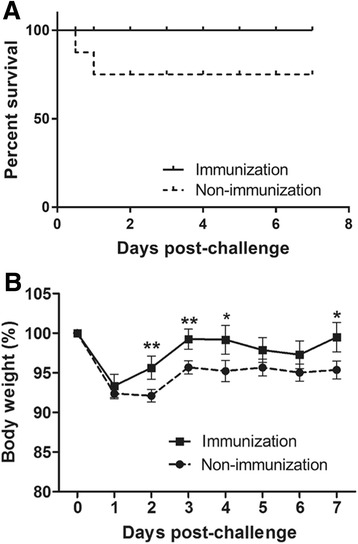
Protective efficacy against challenge with a sublethal dose of JOL654 conferred by JOL1454. **a** Survival rates of immunized and non-immunized mice after challenge. **b** Percentage (%) in mouse body weight. Each point represents the mean of 9 mice per group, and error bars indicate s.d. *P < 0.05 or **P < 0.01 when values were compared with the control group

To assess the protective efficacy conferred by JOL1454 immunization, all immunized mice were intraperitoneally injected with a sublethal dose of virulent STEC JOL654 at week 7 PI. The survival rates and weight losses of the mice were monitored in immunized and non-immunized animals for seven days after challenge. In both the immunized and the control group mice, weight loss, which began only following the challenge, was observed through day 1 post-challenge. While the weights of the immunized animals rapidly recovered, body weight of the negative control was markedly dropped compared to the immunized mice at days 2, 3, 4 and 7 post challenge (*P* <0.05) (Fig. 7b). All immunized mice survived during the entire observation period, but 22.2% of the non-immunized mice died within 18 h of challenge (Fig. 7a). Clinical signs such as diarrhea, hunched posture, and hair election were also observed in non-immunized mice.

## Discussion

Stx2eB is not only an intrinsically non-toxic and immunogenic protein of STEC but also a proven candidate vaccine antigen for prevention and control of ED in pigs [34]. In this study, we constructed an autolyzed *S*. Typhimurium strain expressing Stx2eB, which we designate as JOL1454, and confirmed the expression of Stx2eB fused with the six transmembrane domains (TMD) of the *E. coli* outer membrane protein A (OmpA) in this strain (Fig. 1). OmpA signal peptides have been successfully employed to translocate target antigens across the cytoplasmic space or insert proteins into the outer membranes of gram-negative bacteria [35]. In the present study, detection of Stx2eB protein expression in the cell pellet indicated that the OmpA signal peptide efficiently directed the expressed Stx2eB in the *Salmonella* ghosts (Fig. 1). Additionally, the relative amount of the His-tagged Stx2eB protein was measured in the lysed JOL1454. The significantly elevated levels of antibodies specific to Stx2eB in mice immunized with JOL1454 (Fig. 2) demonstrated that modified *S.* Typhimurium mediated efficient expression of Stx2eB on the outer membrane of the *Salmonella* ghosts.

In this study, immunization of mice with JOL1454 markedly elevated their titers of anti-Stx2eB IgG and sIgA (Fig. 2). The in vivo role of systemic IgG (sIgG) in neutralizing Shiga toxin has been emphasized in strategies to protect piglets against ED [36]*.* Stx-specific sIgG antibodies can protect immunized pigs against systemic infection with STEC, and the magnitude of protection relies on the dose of the antibodies [37]. sIgA antibodies, however, serve as the first line of defense against infection at mucosal surfaces [38]. As the natural infection of STEC occurs via the oral route, sIgA also has an essential role in preventing adherence or attachment of pathogens [39]. These findings indicate that immunization with JOL1454 may mediate protection against ED by eliciting humoral and mucosal immune responses against Stx2eB.

The IgG isotypes, IgG1 and IgG2a, are regarded as markers of T helper (Th) 2- and Th1-type immune responses, respectively [40]. The Fc portions of the IgG1 and IgG2a antibodies primarily interact with the activatory Fc-γ receptors (FcγR) III and FcγR IV, which elicit Th2- and Th1-type immune responses, respectively [41]. The IgG1-to-IgG2a (IgG1/IgG2a) ratio has been used to determine the relative contribution of Th2- versus Th1-type immune responses to STEC infection [42]. In our current study, the IgG1/IgG2a ratio declined from 2.79 to 1.66 in response to JOL1454 immunization (Fig. 3). The decreased ratio indicated that the contribution of IgG2a antibodies continuously increased during the observation period (Fig. 3). This implies that, despite the predominance of the Th2 subpopulation, the Th1 immune response was steadily elevated in JOL1454-immunized animals.

Naïve CD4 + T lymphocytes stimulated with an antigenic peptide differentiate into Th1 and Th2 subpopulations [43]. Th1 and Th2 cells promote cell-mediated immune responses involving antigen-specific cytotoxic effects and humoral immune responses, respectively [44]. JOL1454 immunization significantly elevated the CD3^+^CD4^+^ T cell subpopulations in mice, (Fig. 4a), indicating that JOL1454 can stimulate naïve CD4^+^ T cell maturation. Additionally, we observed that the copy numbers of IL-4 and IFN-γ mRNA were significantly elevated in restimulated splenic lymphocytes of the immunized mice (Fig. 5). The immunomodulatory cytokines, IFN-γ and IL-4, are exclusively expressed in mature Th1 and Th2 cells, respectively [43]. Thus, this observation supports the interpretation that JOL1454 was able to efficiently stimulate CD4+ T cell differentiation into Th1 and Th2 subpopulations, which drive the induction of cellular and humoral immune responses, respectively.

*Salmonella* ghosts elicit robust cell-mediated immune responses (CMI), as they retain the entire surface antigenic determinants of their pre-lysed condition, in the native forms thereof [10]. We observed markedly enhanced in vitro SI in re-stimulated splenic T cells of JOL1454-immunized mice (Fig. 4B). This implies that JOL1454 can induce lymphocyte proliferation, which is a primary parameter of CMI responses. In parallel, mRNA levels of IFN-γ, a marker of CMI, were significantly elevated in splenic T cells after in vitro restimulation. In a previous report, mice immunized with purified Stx2eB protein did not show increased serum levels of IFN-γ [36]. These results support the conclusion that the *Salmonella* ghost system delivering Stx2eB can induce CMI responses in immunized mice.

Ren et al. constructed a plasmid expressing Stx2eB for use as a candidate vaccine, and investigated its protective efficacy using the construct in a mouse model [45]. In that study, immunized mice were intraperitoneally challenged with a virulent ED-associated *E. coli*, and 80% of the immunized mice survived. Immunization of mice with JOL1454 in our study also offered highly effective protection against a challenge with wild-type STEC. While no mortality occurred in any of the immunized mice, approximately 30% of the mice in the non-immunized group died. The protection conferred by JOL1454 immunization might be due to the following: JOL1454 *Salmonella* ghosts efficiently secreted Stx2eB in the cells due to fusion of the protein with *ompA ss*; Stx2eB delivered by JOL1454 ghosts elicited circulating antibodies specific to Stx2eB, which may neutralize Shiga toxin; and *Salmonella* ghosts bearing Stx2eB can trigger CMI responses mediating phagocytosis and elimination of the challenge strain. Additionally, JOL1454 efficiently induced upregulated expression of immunomodulatory cytokines involved with activation of Th1, Th2 and Th17 cells in the pulsed porcine PBMC (Fig. 6), which implicated that JOL1454 may have a capacity to differentiate naïve porcine lymphocytes toward matured CD4^+^ T cell subpopulation. Particularly, the increased expression of IL-6, IL-17A and IL-21 in the present study indicated Th17 cells was matured and activated in the pulsed porcine lymphocytes, which is crucial for intestinal mucosal host defense [46]. These preliminary data supported that JOL1454 has a potential to elicit subsequent immune responses following the cytokine production in porcine.

## Conclusions

The present data indicate that Stx2eB delivered by a *Salmonella* ghost strain could effectively stimulate the immune system so as to confer sufficient protection to prevent ED. The results also suggest that *Salmonella* inactivated by *E* gene-mediated lysis that preserves their intact antigenic determinants may not only effectively deliver the target antigen but also provide an adjuvant property. Additionally, given the immunostimulatory effect of JOL1454 which can elicit immunomodulatory and proinflammatory cytokines in the porcine lymphocyte, this autolyzed *Salmonella* strain producing Stx2eB may constitute a novel alternative approach to developing an effective vaccine candidate against porcine edema disease.

## Abbreviations

BGs: Bacterial ghosts
C_T_: Cycle threshold
DAP: Diaminopimelic acid
ED: Edema disease
ELISA: Enzyme-linked immunosorbent assays
FACS: Fluorescence-activated cell sorting
Gb4Cer: Globotetraosylceramide receptor
Ig: Immunoglobulin
IL: Interleukin
MCS: A multiple cloning site
MTT: 3-(4,5-dimethylthiazol-2-yl)-2,5-diphenyltetrazolium bromide
NB: Nutrient broth
OD: Optical density
ompA ss: Outer membrane protein A signal peptide
PBMCs: Peripheral blood mononuclear cells
PBS: Phosphate-buffered saline
PCR: Polymerase chain reaction
PI: Post-immunization
*S.* Typhimurium: *Salmonella* Typhimurium
SDS-PAGE: Sodium dodecyl sulfate-polyacrylamide gel electrophoresis
STEC: Shiga toxin-producing *Escherichia coli*
Stx2eA: Toxic A subunit of Stx2e
Stx2eB: Homo-pentameric B subunit of Shiga toxin 2e
